# Frédéric Cuvier, a pioneer of comparative psychology in the golden age of French zoology

**DOI:** 10.3389/fpsyg.2026.1815048

**Published:** 2026-04-10

**Authors:** Richard W. Burkhardt

**Affiliations:** Department of History, University of Illinois at Urbana-Champaign, Champaign, IL, United States

**Keywords:** comparative psychology, ethology, evolution, Frédéric Cuver, inheritance of acquired characters, Pierre Flourens, zoology, zoos

## Abstract

Frédéric Cuvier, younger brother of France’s famous zoologist and comparative anatomist, Georges Cuvier, was the first zoologist to seek to establish an institutional science of animal psychology. He did so in his tenure (1803–1837) as superintendent of the menagerie of the National Museum of Natural History in Paris, the first public, metropolitan zoo. That setting simultaneously inspired and constrained his efforts. While the Museum’s zoologists worked with dead specimens, Cuvier sought to expand zoology’s purview and make the menagerie a legitimate site for advancing science. He aimed to study the mental faculties and behavior of the menagerie’s mammals, creating a comparative psychology that would inform human psychology and further the art of training animals. He insisted on a distinct and unbridgeable gap between the human and animal mind, and he rejected the idea of evolution, but he championed the concept of the inheritance of acquired characters. His efforts were hampered by his animal subjects’ cramped conditions of existence, his subordinate position at an institution that prioritized dead specimens, the multiple responsibilities of his post, and other work that took his attention away from behavioral research. His work compares with the work of twentieth-century ethologists and comparative psychologists in diverse and interesting ways. This paper emphasizes the value of understanding Cuvier in his own historical context but also suggests that when comparative psychologists look for early pioneers in their field, Frédéric Cuvier’s credentials outshine those of Pierre Flourens.

## Introduction

In our recent book, *The Leopard in the Garden: Animal and Human Lives in Paris at the First Public Zoo of the Modern Era*, we extensively outlined Frédéric Cuvier’s scientific biography (Burkhardt, 2026). In the present article, we will highlight the major features of Frédéric Cuvier’s scientific path, focusing on the aspects most relevant to animal behavior, and we will show how he can be considered an early pioneer of comparative psychology. Sources for the mentioned historical facts and quotations are contained in Burkhardt (2026). All quotations in this paper are translated by the author from the original French. Quotations cited as “in Burkhardt” are from unpublished manuscripts in the Archives Nationales de France and are fully referenced in Burkhardt (2026).

**Figure 1:**
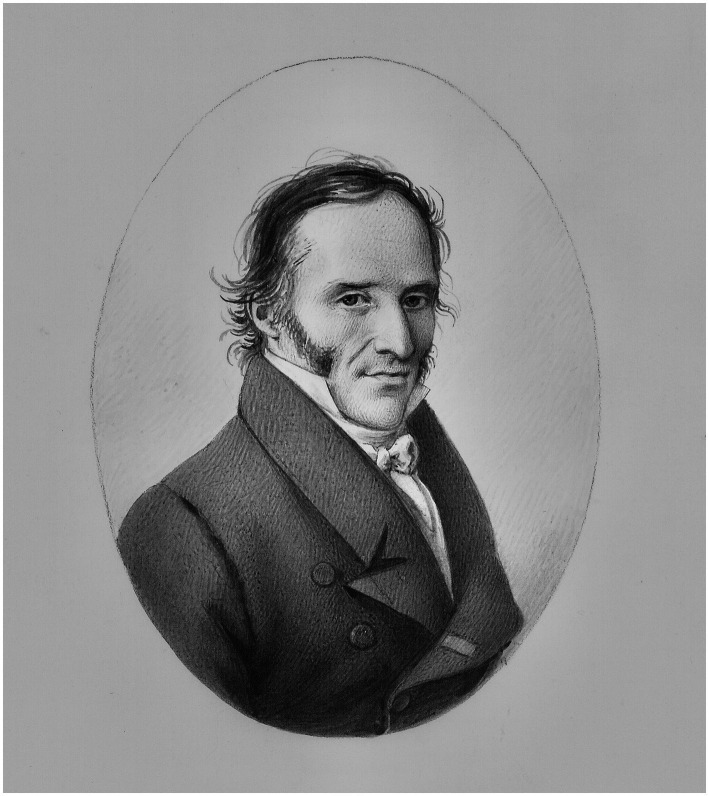
Frédéric Cuvier, as portrayed in 1826 by Antoine Chazal (1793–1854). Source: Muséum national d’histoire naturelle. MS 2671–21, fol. 3. Reproduced with the permission of the Muséum national d’histoire naturelle.

Before science was widely professionalized, before academic zoologists recognized comparative psychology (or animal behavior) as a subject worthy of a professorial chair or any other institutional position, Frédéric Cuvier (1772–1838), the younger brother of France’s leading zoologist and comparative anatomist, Georges Cuvier (1769–1832), sought to create an institutionalized science of comparative psychology / animal behavior. He did so as superintendent of the menagerie of the Muséum national d’histoire naturelle in Paris, a post to which he was appointed in 1803. Thirty-four years later, his career was capped by promotion to a new professorship at the Museum, but it had not come easily. His brother had told him it would be improper for them to be professors at the same time. Two months after Georges’ death (in 1832), Frédéric urged the Museum to petition the government for a chair on “the nature of living animals and their training.” The Museum postponed deciding, then shelved the request altogether. Cuvier renewed his efforts in December 1836, this time going directly to the minister of public instruction and asking for a chair on “the psychological nature of animals.” The minister, François Guizot, similarly called it “a chair on the psychology of animals” when he solicited the Museum’s opinion of it. The preceding quotes are all in Burkhardt (2026). The professors preferred considering it as a chair of “comparative physiology,” but some doubted whether there was enough yet known to constitute a science. In February 1837 they rejected the chair by a 7–5 vote. Ten months later, a new minister of public instruction, Nicolas Salvandy, brought Cuvier’s proposal back to them. This time they acquiesced (10–1, with one abstention). Cuvier was awarded the new chair of “comparative physiology,” with the menagerie under its aegis. “Psychology” was not in the title, but it was clear Cuvier’s research and teaching would focus on animal mental faculties and behavior. as well as on the application of this knowledge to animal training (Figure 1).

### Frédéric Cuvier at the menagerie

Nothing about Frédéric Cuvier’s early life suggests he would have ended up in a career devoted to studying living animals. Unlike many comparative psychologists and ethologists of the twentieth century (Dewsbury, 1989), he never testified to having been an animal lover in his youth. Nor as a young man did he evidence any special aptitude or interest in a career in science. He was embarked on the life of a watchmaker when his brother called him to Paris in spring 1798 to be his multipurpose assistant. This introduced Frédéric to science in Paris, but it did not induce him to become a comparative anatomist or zoologist like Georges. When he decided he might 1 day be able to contribute to science, the field he chose first was chemistry.

Five-and-a-half years after his arrival in Paris, Frédéric Cuvier’s prospects were dramatically altered by being appointed superintendent of the Museum’s menagerie. The niche was unique. The post was entirely new, and the menagerie (just 10 years old) was keen to portray itself as new too, wholly different from the royal menagerie at Versailles with all its extravagance and frivolity. It aimed to serve the needs of the new republic, not the vanity of aristocrats. It was the first public, metropolitan menagerie, the first “zoo” of the modern era. It represented a major cultural innovation, a model for other new zoos throughout the world (the London Zoo, founded in 1828, was the first to follow). To understand Cuvier’s career in his new post and how he fared with respect to his goals, one must consider the institutional, material, and intellectual conditions of his existence (Burkhardt, 1999).

When Cuvier was made superintendent, the menagerie’s prospects were just beginning to look secure. Its origins had been neither planned nor propitious, and in its early years it ricocheted between moments of glory and threats of immanent ruin. It came into being via contingent events in November 1793, at the height of the French Revolution, when animals exhibited on the city streets were confiscated by the police and delivered to the new National Museum of Natural History (founded 5 months earlier). Depending entirely on government funding, the Museum and menagerie’ fortunes were tied to the Republic’s. In the late 1790s, as the government and city groaned under severe shortages, the Museum feared the menagerie’s animals would die from starvation. However, circumstances improved early in the new century. The menagerie’s grounds were expanded, its animal population grew, and the site became an attraction on the itineraries of visitors to Paris (having already been a draw for the Parisian populace from the start). In the summer of 1804, when Cuvier tallied the menagerie’s animal population, it had more than 100 mammals and nearly as many birds. Among the mammals were 20 big cats representing six different species, three species of bears, an elephant, two camels, three dromedaries, multiple species of deer and sheep, a gnu, and more. The birds included eagles, vultures, two ostriches, and a cassowary.

Yet what attracted public curiosity and what was important to science were not identical, and the second remained an open question. Previously, menageries had served science primarily by providing fresh cadavers to anatomists and taxidermists. The value of *living* specimens was yet to be demonstrated. The possibilities identified in the 1790s included studying animals’ physiology, reproduction, and developmental and seasonal changes, plus their potential for domestication, acclimatization, and even hybridization. No one, however, was championing the new menagerie as a site for studying animal behavior. The eighteenth-century’s leading authorities on animal life—Georges Louis Leclerc, comte de Buffon; Hermann Samuel Reimarus; and Charles-Georges Le Roy—had all maintained that the behavior of animals in captivity was too constrained and distorted to help understand how animals behave in nature.

The menagerie’s primary years of development coincided with the golden age of French zoology (late 1790s–1830s), highlighted by the work of Museum professors Georges Cuvier, Jean-Baptiste Lamarck, and Étienne Geoffroy Saint-Hilaire, who developed and exploited the burgeoning collections in their respective fields. They disputed matters of theory, but their practices were essentially the same. They were all “cabinet” or “sedentary” naturalists who worked with dead specimens, studied their form and organs, compared them with other specimens (and descriptions and illustrations), and sought to arrange them according to a natural order. They benefited from the efforts of voyager naturalists who collected specimens abroad. Significantly, Georges Cuvier instructed voyagers not to expend time, effort, and funds pursuing live specimens when dead specimens were what the Museum needed most. Though the menagerie thrived in French zoology’s golden age, it was never the top priority of the Museum’s zoologists. Frédéric Cuvier did the best he could under the circumstances.

Another constraint on the younger Cuvier’s efforts to build a science of animal behavior were his main responsibilities as menagerie superintendent. The post’s primary purpose was not the advancement of further scientific knowledge. In 1803, what the Museum needed above all at the menagerie was someone to ride herd over the animal keepers, who, left to their own devices, flaunted the Museum’s rules. The administration was scandalized by how the keepers solicited bribes from a public willing to pay for entry on days or at times of day when the menagerie was officially closed. Beyond keeping the keepers in line, the superintendent was charged with overseeing the food supplies and the animals’ feeding, the care of ill animals, maintaining sanitary conditions, and more. On the more scientific side of things, the superintendent was instructed to study the animals’ suitability for domestication and acclimatization and to observe such aspects of the animals’ lives as their periods of rutting, gestating, laying, etc. One of the regulations defining the post charged the superintendent with conducting experiments according to a program the zoology professors were to give to him, but they actually never produced one for him. He was left to his own initiatives to find new ways of advancing science. Creating a science of animal behavior based on the menagerie’s animals was his own idea. He also proposed the menagerie as a site for creating a science of variation and heredity, saying this would illuminate both the issue of species mutability and the art of animal breeding.

In addition to the above-mentioned challenges, Frédéric Cuvier had to negotiate the shifting friendships, alliances, and enmities of the Museum’s institutional ecology. They helped in some instances and hurt in others. Personal connections secured him the menagerie post in the first place. Étienne Geoffroy Saint-Hilaire, the professor of mammals and birds, steered his colleagues’ choice to Frédéric, though some had favored a veterinarian for the job. Geoffroy argued that Frédéric was well-informed in natural history, skilled at bookkeeping and managing people, and known to the Museum as a man of excellent moral character. What Geoffroy did not mention was his hope that securing the post for Frédéric would help Geoffroy reestablish the close friendship he had with Georges Cuvier from 1795 to 1798, before Geoffroy left Paris to take part in Napoleon’s expedition to Egypt. Returning 4 years later, Geoffroy found that George’s scientific career had skyrocketed and the magic of their earlier years was hard to recreate. When Geoffroy engineered Frédéric’s new appointment in 1803, he did not foresee that a quarter of a century later he and Georges would be rivals and Frédéric would appear to him as an ingrate seeking to undermine his authority. Geoffroy would end up complaining recurrently to his colleagues about Frédéric’s insubordination. Frédéric, for his part, felt stuck under a boss increasingly unsympathetic to him and his efforts.

**Figure 2:**
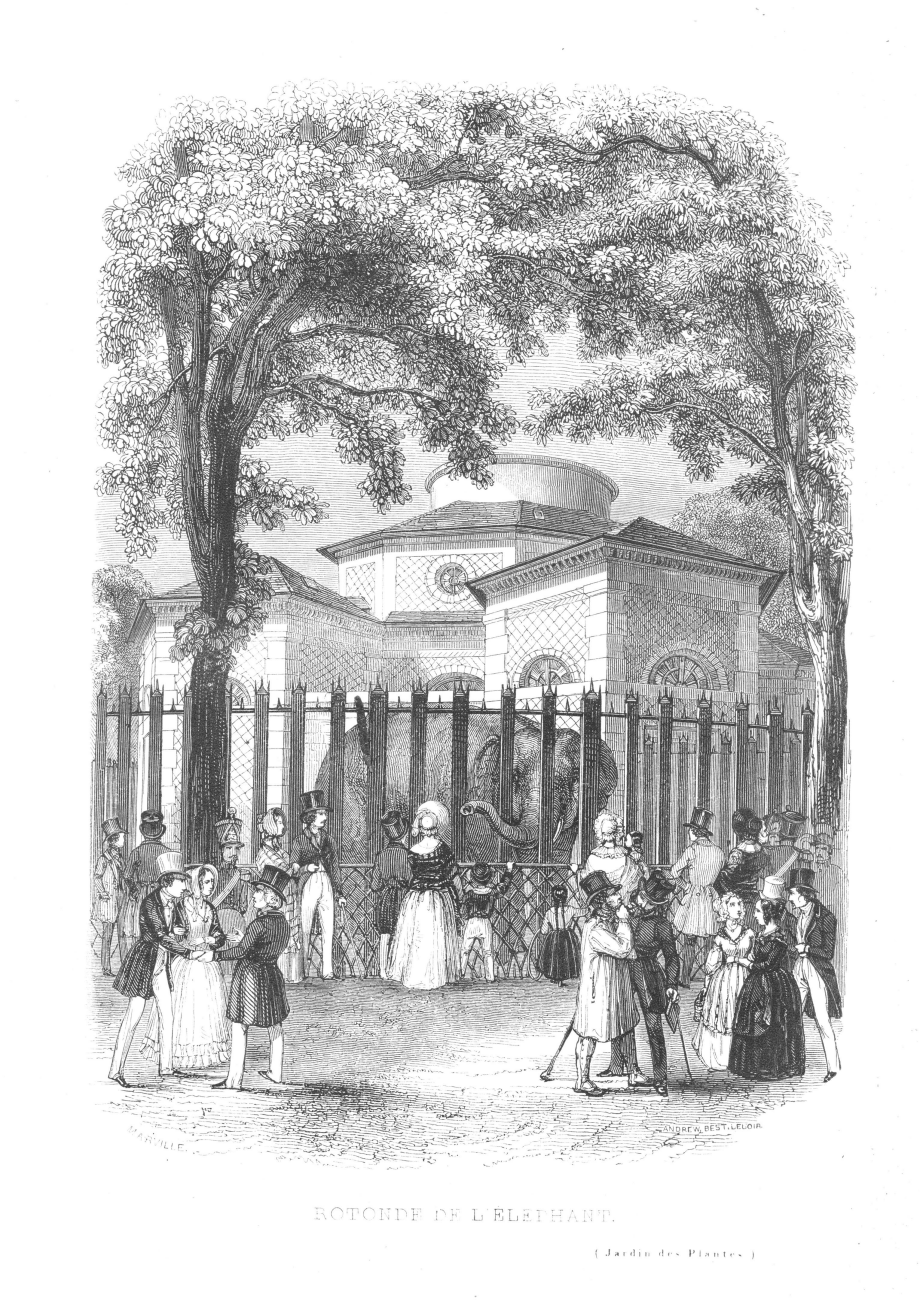
The public visiting the elephant in its park at the rotunda of the menagerie. Engraving by the atelier Andrew, Best, & Leloir, after an image by Charles Marville (1813–1879). From: François Boitard, *Le Jardin des Plantes* (1842). (Boitard and Janin, 1842).

Setting to work at his new job in January 1804, Frédéric Cuvier was immediately challenged by the tasks of managing the keepers and providing for ill animals, several of which died in his first weeks. In a menagerie guidebook he produced that summer, he lamented how little was known about feeding exotic animals, caring for them in a climate hostile to them, and treating their diseases. Despite these challenges and the realities of the animals’ cramped conditions, he constructed a bold claim about the menagerie’s scientific potential. In his first scientific paper, he wrote: “Menageries can be for zoologists what the laboratory of the chemist is for those who study inorganic bodies” (Cuvier, 1807, p. 119). In later writings, he elaborated on this, saying that just as controlled experiments had been key to advancing the physical sciences, so too could they advance the study of animal faculties better than observations in nature could (Anon and Cuvier, 1809). His promises about experimentation notwithstanding, however, his behavioral work would depend more on observations in the menagerie than experiments there (Figure 2).

### Instincts vs. learned behavior, the inheritance of acquired characters, and Cuvier’s first mention of “comparative psychology”

Cuvier’s first sketch of his plans for behavioral studies came in 1808 in a paper on the dog of the Australian aborigines. In introducing the paper with “some reflections on the moral faculties of animals,” he identified for himself the task of establishing “with precision” the respective roles of instinctive and acquired elements in the behavior of different animal species (Cuvier, 1808, pp. 459–460). Even while distinguishing instincts from learned behavior, he explained they were interrelated. Actions learned through training, he claimed “become finally instinctive or hereditary as soon as they have been exercised over a series of sufficient generations.” Conversely, he said, when such actions stopped being exercised, they gradually wear away (Cuvier, 1808, p. 462).

Cuvier thus endorsed the idea today known as “the inheritance of acquired characters.” For biologists today, the concept is inextricably linked with the name of Lamarck, the primary evolutionary theorist before Darwin, but Cuvier never felt the need to credit Lamarck with it, nor did he even mention Lamarck’s embrace of it. Furthermore, Cuvier wholly rejected the idea of species transformation. Though he viewed the long-term effects of use and disuse as a major cause of intraspecific variation, he was convinced these variations were limited and would never proceed far enough to produce a new species. He attributed the idea of the inheritance of acquired characters to the eighteenth-century naturalist Charles-Georges Leroy. It seems nonetheless to have been Cuvier himself who, in discussing the idea, first used the terms “hereditary” and “heredity” in their modern, biological sense (Gayon, 2006; Burkhardt, 2011).

The inheritance of acquired characters was key to Cuvier’s first invocation of “comparative psychology.” Discussing the Australian aborigines’ dog, he predicted that dogs domesticated for only a few centuries would have different mental characteristics than dogs domesticated over several millennia. He proposed comparing the mental faculties of the different dog races of “barely civilized people,” i.e., the dogs of “Australia, Patagonia, New Zealand, Siberia, Lapland, Iceland, etc.,” as a means of illuminating “the successive development of the intelligence of their species.” This, he continued, would provide “the means of constructing their comparative psychology [and] would lead us to precious results, even for the psychology of man” (Cuvier, 1808, p. 470). This was his first use of the phrase “comparative psychology,” comparable in antiquity to Jean-Baptiste Nacquart’s use of it, identified by Raffaele d’Isa as the phrase’s first appearance in French (d’Isa and Abramson, 2023). What Cuvier proposed in 1808 for the different races of one species, the dog, was a mini version of his broader plan for a comparative psychology of all mammals.

### The gap between the animal and the human mind, and the specter of evolution

Cuvier’s next important foray into behavioral issues was a paper detailing his observations of a young female orangutan, the first ever brought to France. Cuvier studied her not at the menagerie (it did not receive its first orangutan until 1836), but instead at the home of a man who was nursing her back to health prior to delivering her to the Empress of France Josephine Bonaparte. Highlighting the “astonishing phenomena” (Cuvier, 1810, p. 56) that he witnessed there was evidence that the orangutan was not only able to learn through trial and error but could also generalize new ideas from previous ones. For instance, in one case, she moved a chair to station it beside a door, climbed the chair, reached the door latch, and opened the door. She had never seen anyone perform this combination of actions, but she knew that chairs were moveable, and she had previously discovered on her own that when a chair was beside the door, she could climb it, reach the latch, and open the door. Wrote Cuvier, “How could one not recognize in this action the faculty of generalizing? … I do not believe that any other animal has carried the force or reasoning further.” (Cuvier, 1810, p. 62). He was also impressed by how, in moments of pique, after being refused something she wanted, “she struck her head on the ground as if to excite interest or pity” and then “lifted her head from time to time and suspended her cries to look at the people nearby and see if she had produced any effect on them ….” (Cuvier, 1810, p. 61). Confident from these and other of his observations of the “animal that comes closest to man,” Cuvier predicted it would not be difficult to make observations and experiments on “on the intellectual characters that distinguish one species from another” over the whole class of mammals (Cuvier, 1810, p. 65; Burkhardt, 2018).

But it was not easy to learn much from animals that lived in cramped quarters and/or survived only briefly. For example, of the three seals that arrived at the menagerie in 1809 (two in the summer, one in the fall), not one lived to the year’s end. Cuvier’s plans of studying them were hampered by how young they were, how hard it was to find food they would eat, and “the impossibility of making them live long enough for sustained experiments” (Cuvier, 1811a, p. 397).

Cuvier had more luck with several beavers, the first of which arrived at the menagerie in 1811. He found their intelligence much overrated. Buffon had credited beavers with the faculty of “understanding one another and acting in concert.” In their communal action constructing dams and lodges, wrote Buffon, beavers display “a glimmer of intelligence, which, although very different from that of man in principle, produces rather similar effects” (Buffon comte de, 1760, p. 285). Cuvier concluded to the contrary that the species’ famous efforts were a function of instinct rather than intelligence. He gave his beavers willow branches, straw, and earth and watched how they piled these between the bars and shutter of their cage. He found that they worked independently rather than in concert. That instinctive nature of the beaver’s impulse to build was further evidenced by the actions of a specimen that had been captured as a kit and had never had the chance to see other beavers build, because its parents had simply lived in a hole in a riverbank (Cuvier, 1817).

Cuvier’s beaver studies were not a threat to contemporary thinking, but the intellectual powers he ascribed to the orangutan were. Someone (likely his brother) apparently took Frédéric aside after his orangutan paper’s publication and, we surmise, underscored the potential religious, political, and/or philosophical dangers of failing to insist on a distinct and unbridgeable gap between the human and the animal mind. Frédéric had not suggested the orangutan’s intellectual powers equaled a human’s, but neither had he taken care to declare that the mental gap between humans and animals was clear and inviolable. Evidence for close proximity threatened the belief in human uniqueness. Contemporary evolutionary thought exacerbated the situation. In 1802 in his *Recherches sur l’organisation des corps vivans*, Lamarck summarized the physical features distinguishing humans from apes and asked, “could not one think that this particular state of human organization *has been acquired little by little after much time, aided by favorable circumstances*? What a subject of meditation for those who have the courage to dive into it!” (Lamarck, 1802, p. 134). Later, in his *Philosophie Zoologique*, Lamarck revisited the question, speculating on how a species like the chimpanzee (the “orang of Angola”), under the right conditions and with an immense amount of time, could come to walk upright on two feet, develop new needs and habits in its communication of ideas, develop the faculty of speech, and acquire a bodily organization identical to a human’s (Lamarck, 1809).

In this context, it behooved Cuvier to insist on an unbridgeable gap between the animal and the human mind. He did so in his next paper on animal mental faculties, saying only humans had the ‘faculty of meditating, of reflecting” (Cuvier, 1813, p. 217). Later, in a long essay on instinct, he wrote that establishing “the presumed point of separation between the intelligence of the human species and the intelligence of animals” “ought to be the principal goal” of the kinds of studies he was promoting (Cuvier, 1822, p. 536). Further on in the same essay he specified where he perceived the difference to be: “The human species, unique to all others, has the faculty of being enlightened, of acquiring pure ideas, of making them the prototype of justice, beauty, and truth, and of working toward its improvement: this is its true prerogative, and it owes this to the faculty of self-knowledge and reflection” (Cuvier, 1822, p. 543). Two years later, in a general essay on the orangutan, he repeated most of his 1810 orangutan paper word-for-word, including his descriptions of the animal’s behavior, but he quietly excised the places where he credited the orangutan with the ability to generalize. He did not disown his earlier appraisal; he just stopped mentioning it. He also omitted an introductory section of the paper that situated the orangutan’s mental faculties with respect to those of other animals (Cuvier, 1824). In 1832, drawing up an overview of the public lecture course he would teach if he became professor, he made clear he wasn’t threatening the moral order. The first section, on the general nature of animals, would distinguish between their “cognitive, instinctive, and organic” faculties. This material, he said, “will lead us to the absolute distinction that separates man from the animals.” In the same section, he would “show, with some details, the care nature takes to establish the most perfect harmony between [animals’] physical and intellectual faculties,” and further how their place in the economy of nature depended on their exercise of these faculties, all of which manifested “the wisdom and depth of the views of Providence” (Legée, 1992, p. 142; Burkhardt, 2026, p. 637). A copy of Cuvier’s course program was found among his papers after his death and published in *Annales des Sciences Naturelles (Zoologie*; Cuvier, 1839).

Frédéric Cuvier had not invoked “Providence” in his scientific papers. Very sparingly, he gave a nod to the “author” or “first cause” of things, but his mention of “Providence” in his course proposal was a more explicit embrace of a creationist world view. His reticence in his scientific papers was not from a lack of conviction, but rather because he, like his brother, considered the matter of final causes outside science’s purview.

Georges Cuvier thought the best way to handle speculative “systems” like Lamarck’s was simply to ignore them, but when confrontation became necessary, he offered scientific, not religious objections. He argued that animal structures and actions were so closely correlated to each other and the conditions of their existence that any change would render an animal unable to survive. In his *Recherches sur les ossemens fossiles* (Cuvier, 1812), he pointed further to the lack of transitional forms between fossil and modern species, the identity between modern species and their centuries-old counterparts recovered from the tombs of ancient Egypt, and the limits to variation in wild and domestic species, citing for the latter his brother’s study of osteological differences between different varieties of dogs (Cuvier, 1811b).

Frédéric Cuvier, for his part, thought the menagerie could be a new site for addressing the species question. He suggested this in 1815 when urging the Museum to turn the former elephant park into a park for experiments (Burkhardt, 2001). He repeated it in his request for a professorship, saying his work on animal faculties would apply “to zoology, to give to the idea of species a precision it does not have” and also to the art of training animals to make them serve our needs” (Cuvier, F., unpublished letter to the professors of the Museum, 28 July 1832, Archives Nationales de France, AJ/15/644). Addressing the species question and the training of animals each required attention to the variation of habits and forms. In the first, he hoped to establish the limits to variation. In the second, he was interested in how variations induced by training could improve animals with respect to human needs.

### Improving animals via training and breeding

According to the teaching plan Cuvier presented to the Museum’s professors in 1832, Section 2 of his course would illuminate the roles of instinct and learning throughout the whole mammal class. This would facilitate identifying the species eligible for domestication and further development, the subject of Section 3, “on the training (*éducation*) of animals.” Cuvier had already written on the subject in two long papers on animal sociability and domestication (Cuvier, 1825b; Cuvier, 1825a). Where Buffon had represented the domestication of animals as a “conquest,” Cuvier saw it instead as a “seduction.” The only species susceptible to domestication, he insisted, were those that were social, lived in troops, and formed attachments. Their seduction involved enhancing and satisfying their original needs and then developing newly acquired habits further. Breeders were aided in this project, Cuvier explained, by “one of the most general laws of life: the transmission of organic or intellectual modifications by way of generations” (Cuvier, 1825b, p. 454).

Significantly, Cuvier recognized two different kinds of heritable modifications. On the one hand, there were the mental or structural changes produced when new needs led to new habits. On the other, there were what he called “accidental” (Cuvier, 1811b, p. 349) or “fortuitous” modifications. He used the latter adjective when in hailing “one of the most astonishing phenomena of nature: the transformation of a fortuitous modification into a durable form, of a passing need into a fundamental propensity, of an accidental habit into an instinct.” (Cuvier, 1825c, p. 454). A dozen years later, the young Charles Darwin read an English translation of Cuvier’s paper and copied Cuvier’s comments on “fortuitous” variations into his B notebook on transmutation. This may have been what initially focused Darwin’s attention on the idea of “fortuitous” variations (De Beer, 1960). Later, Darwin would take the momentous step of viewing the “natural selection” of fortuitous variations as the primary mechanism of species change. Frédéric Cuvier, however, like others familiar with breeders’ achievements, could not imagine that step. Knowing that breeders’ efforts had never produced a new species, Cuvier remained confident in species fixity.

### Frédéric Cuvier and ethology

Cuvier’s goals for a science of animal behavior were constrained by multiple factors. The foremost was what Cuvier identified with some bitterness in 1833 as “the arrangement of the menagerie never having been conceived with the view of facilitating the study of the native disposition of animals” (Cuvier, 1833, p. 3). The Museum prioritized dead specimens over live ones, and the live animals, locked in cages, could not display the behavior of which they were capable. Geoffroy Saint-Hilaire, the professor of mammals and birds, was proud to have the menagerie as his charge, but he regarded the public’s entertainment, not the advancement of science, as the menagerie’s main purpose. Furthermore, Geoffroy by the 1830s was not interested in helping Frédéric. Geoffroy’s friendship with the Cuvier brothers had soured years earlier, he had clashed mightily with Georges on scientific matters, and he had complained recurrently to his colleagues about Frédéric’s insubordination. Georges Cuvier’s death in 1832 did not alleviate the tensions between Geoffroy and Frédéric regarding the management of the menagerie. Their unhappiness with each other was expressed anew at the Academy of Sciences in spring 1837 when Geoffroy accused Georges of denying species mutability out of political expediency and Frédéric sharply defended his brother’s scientific integrity (Burkhardt, 2026).

But even if Cuvier had had better facilities and better relations with Geoffroy, the challenges of studying instincts and learning in mammals would have been immense. And then too there was the problem of having the time for it. Cuvier had to spend countless hours in his duties as superintendent, and he also devoted time to a whole retinue of mainstream anatomical and classificatory studies (e.g., Cuvier, 1836; Cuvier, 1825c; Cuvier, 1811b) and months to his administrative work for the University of France. Eventually time ran out on him. In July 1838, visiting Strasbourg on university business, he succumbed to a sudden illness. In his 7 months as professor, he had not had the occasion teach his course. Had he done so and assembled his lectures into a book, he would surely be seen today as the founder of comparative psychology, but that synthesis was never achieved.

Might one consider Frédéric Cuvier an early ethologist? Distinguishing between instinct and learning in animal behavior was a fundamental goal of Cuvier’s, as it would be for ethology’s twentieth-century founders, Konrad Lorenz and Niko Tinbergen. Where Cuvier dealt with mammals, they worked primarily with birds, but that does not constitute a fundamental divide between them. They contrasted markedly, though, in their views on domestic animals as scientific subjects, and, more generally, their feelings on domestic versus wild animals. Lorenz considered the instincts of domestic animals weak and disordered compared to those of wild forms, especially with respect to preventing interspecific mating. He viewed the wild form of the species as the ideal. Cuvier, in contrast, hailed the improvements civilized peoples had made in animals by subjecting them to beneficial conditions and selecting the best variations for breeding. In the horse, he said, humans had developed “beautiful races” that nature alone could not have perfected to the same degree (Cuvier, 1837, p. 674). The most fundamental divide between Cuvier and the twentieth-century ethologists, nonetheless, was that he believed in a divinely ordered creation, and they understood animal behavior entirely in terms of evolution. Furthermore, they were neo-Darwinists, confident that the “Lamarckian” idea of the inheritance of acquired characters was unfounded. Knowing of Cuvier’s belief in the inheritance of acquired characters would not have made them feel any more akin to him.

Yet while Cuvier’s guiding assumptions differed from the modern ethologists’ in fundamental ways, they match closely what Isidore Geoffroy Saint-Hilaire, Étienne’s son, had in mind when he became the first to use the words “ethological” and “ethology” in a zoological context. Historians have cited Isidore’s words but without recognizing how closely they mirrored Frédéric’s work (e.g., Jaynes, 1969). In 1854, publishing the first volume in a multi-volume *Histoire naturelle générale des règnes organiques*, Isidore previewed a fourth section of the work that would present “general facts, relations, and *ethological* [italics added] laws pertaining to instincts, habits [*mœurs*], and more generally to the external vital manifestations of organized beings.” The section would begin with a distinction between “intelligence and instinct” in animals and a discussion of “automatic acts.” It would address how the habits of animals serve the preservation of the animal and the species; social species; animal training; “modification of habits, and as a result, of the instincts of domestic animals”; “permanence of acquired instincts;” and more (Geoffroy Saint-Hilaire, 1854–1862, vol. 1, p. xxii). Referring again in volume 2 to the work’s forthcoming fourth section, he wrote, “to ethology … belongs the study of the relations of organized beings in the *family* and the *society*, in the *aggregate* and the *community*” (Geoffroy Saint-Hilaire, 1854–1862, vol. 2, p. 285). Isidore did not name Frédéric Cuvier in these previews, but had he lived long enough to write the section, Cuvier’s name would have featured in it, because he was the leading writer on these topics, and Isidore knew his work very well. Cuvier had had good relations with Isidore even when he had difficulties with his father. At Cuvier’s request, Isidore had substituted for him as menagerie superintendent during six summers in the 1830s when Frédéric was away performing his University of France administrative duties.

Space limitations here do not allow an elaboration of how closely Frédéric Cuvier’s work resembles Isidore Geoffroy’ Saint-Hilaire’s use of the word “ethology” when he introduced it to zoology, but perhaps enough has been said to justify identifying Cuvier as “the first ethologist of the *first* kind” (i.e., Isidore Geoffroy Saint-Hilaire’s kind), the purpose of which is to bring our attention back to Cuvier’s own concerns and their relation to the historical context that inspired them. We review that context in the conclusion in addressing how to think of Cuvier’s aims and aspirations in relation to the history of comparative psychology.

## Conclusion: Frédéric Cuvier and comparative psychology

In French zoology’s golden age, Frédéric Cuvier urged moving zoology beyond its focus on lifeless forms to include the living animal, its faculties, and the behavior dependent on those faculties. He was inspired to do so by his appointment as superintendent of the Museum of Natural History’s menagerie. He sought to make the menagerie a laboratory for controlled experiments on animal instincts and intelligence. The broader goal of this, he explained, was to understand how the mental faculties of animals suited them to the conditions of their existence and their role in the economy of nature. A more utilitarian goal was to advance the art of training animals for human purposes. As early as 1808 he envisioned a “comparative psychology” that would consider the different mental faculties of all the different mammalian genera if not species. In 1829, when Frédéric Cuvier wrote the article for the entry “Zoologie” (Zoology) of the *Dictionnaire des sciences naturelles* (Dictionary of Natural Sciences), an encyclopedia for which he was also the general editor, he underlined how psychology was fundamental to zoology. In particular, he stated that psychology (“psychologie”), together with anatomy and physiology, were “indispensable auxiliaries; without them, all of zoology would be nothing but a vain science, more suited to exercising the powers of the mind than to enlightening it” (Cuvier, 1829, p. 357). Cuvier did not employ the word psychology in the rest of this essay, but his claim regarding the importance of psychology to zoology was crystal clear. He elaborated on this theme when he lobbied in the 1830s for a chair at the Museum. When he wrote in 1836 to the minister of public education, Guizot, about a chair on “the psychology of animals,” he argued that a zoology that studied only the material features of animals and not their mental faculties and actions was a “truncated” science, as incomplete as would be “the study of man [restricted] to his anatomy and physiology.” He urged further that just as human anatomy had been enriched by anatomical studies of animals, so too would human psychology be illuminated “when one brings to bear on it the numerous psychological phenomena [phénomènes psychiques] presented by the long, animal series.” This was especially the case, he said, “because these phenomena have a simplicity that we rarely find in [humans]. In this respect, in effect, animals, in what they have in common with us, offer truly a natural analysis of human intelligence.” These passages from his letter to Guizot reappear in his posthumously published manuscript, *Considerations on the Study of Animal Actions* (*Considérations sur l’étude des actions des animaux*; Cuvier, 1839, p. 141). Further along in his 1836 letter to Guizot, he identified the subject of his course as “the psychological [psychique] nature of animals.” The course would address “the method that must be applied to the psychological phenomena [phénoménes psychologiques] in animals, the actions in which these phenomena manifest themselves in the animal series as well as the laws governing them; and finally, the application of these laws to developing animals, rearing them, attaching them to our species, and training them according to our needs (cited in Burkhardt, 2026, p. 637).

Although the chair Cuvier received late in 1837 was named “comparative physiology,” the program that the Museum drew up for the chair specified that the professor would deal not only with organ function but also “the faculties of intelligence and instinct” and that he would furthermore offer “observations and experiments on animal training” (Museum of Natural History letter to the Minister of Public Instruction, December 20, 1837, Archives Nationales de France, AJ/17/13566). In brief, the chair explicitly had Cuvier’s kind of animal behavior studies in its purview. The direction of the menagerie was also one of its attributes.

After Frédéric Cuvier’s death in 1838, he was succeeded in the chair of comparative physiology by his close friend Pierre Flourens, a disciple of Frédéric’s brother, Frédéric and Flourens were already friends when Flourens’ successes in experimental physiology in the early 1820s brought him wide attention (Legée, 1992). In 1824, Frédéric engaged Flourens to write articles on physiology for Frédéric’s *Dictionnaire des Sciences Naturelles*. A letter Frédéric wrote Flourens the following year shows they were pondering their respective chances for professional advancement, including election to the Academy of Sciences (Legée, 1992). Cuvier was elected to the Academy before Flourens (in 1826 and 1828 respectively), but Flourens was named first to a professorship at the Museum (in 1832, to the chair of human anatomy). In December 1836, Flourens was part of a three-person committee at the Museum appointed to assess Cuvier’s request for a chair of animal psychology (Burkhardt, 2026). It seems probable that Flourens knew Frédéric Cuvier’s work and aspirations as well as anyone did. In any case, after Cuvier’s death, Flourens took it upon himself to make sure that his friend’s work was not forgotten. Flourens surveyed Cuvier’s writings in depth in a series of three lectures to the Academy of Sciences in 1839, which he published the same year in the Academy’s *Journal des savants*, in the *Annales des sciences naturelles*, and as a stand-alone monograph (Flourens, 1839a, 1839b, 1839c). This work became the basis of his book, *Analytical Resume of the Observations of Frédéric on the Instinct and Intelligence of Animals* (*Résumé analytique des observations de Frédéric Cuvier sur l’Instinct et intelligence des animaux*; Flourens, 1841). New editions of this book followed (Flourens, 1845), though in the third and later editions Cuvier’s name was omitted from the title page (Flourens, 1851). In his eloge of Cuvier at the Academy of Sciences in 1840 Flourens also highlighted Cuvier’s behavioral studies, stating that “the most important, the most original” part of Cuvier’s work were “his observations on the instinct and intelligence of animals, observations which interest the philosopher no less than the naturalist, and to which he consecrated 30 years of sustained and conscientious studies” (Flourens, 1840, p. v).

Flourens’ *Instinct and Intelligence of Animals* would have been a much better candidate for the title *Comparative Psychology* (*Psychologie comparée*) than the book that Flourens gave that name almost a quarter of a century later (Flourens, 1864b). The latter title appears to have contributed decisively to Flourens’ reputation as a pioneer in comparative psychology, but a modern comparative psychologist reading this book will be surprised and disappointed to find that only about a quarter of the text is about the instincts and intelligence of animals, and none of that involves original work by the author. By Flourens’ account, *Comparative Psychology* was the second edition of a book first entitled, *On Reason, Genius, and Insanity* (*De la raison, du génie et de la folie*; Flourens, 1864a). Flourens decided *Comparative Psychology* was the proper title, but the contents represent the first title more accurately. The phrase, “comparative psychology” goes unexplained in the book other than to say that the instincts of animals make up “a very considerable part of comparative psychology” (Flourens, 1864b, p. 161).

Flourens’ scientific work made him admirably suited for the Museum’s chair of comparative physiology, but his research was decidedly different from Frédéric Cuvier’s. Whereas Cuvier’s approach was to study animals through “observing them and submitting them without physical suffering to methodical experiments” (Cuvier, 1839, p. 142), Flourens gained fame for the vivisection studies where he surgically ablated different parts of the brain, central nervous system, etc., to determine their functions (e.g., Flourens, 1824). Along with many other physiological studies, he conducted anatomical and pharmacological studies and wrote an important critique of phrenology. However, of the 110 scientific papers credited to Flourens in the Royal Society *Catalogue of Scientific Papers* (Royal Society, 1868, 1877), not one represents an original contribution on his part to the psychology of animals, if one defines the phrase as he did in 1864: “The psychology of animals is the study of their instincts and their intelligence: [the] study that immortalized Réaumur … and rendered more or less famous Georges Leroy, Frédéric Cuvier, and the two Hubers [François and Pierre] (Flourens, 1864b; Flourens, 1864a, p. 269). Curiously, Flourens had not used the word “psychology” previously in his book on instinct and intelligence in animals, even though Frédéric Cuvier, whose works were the primary basis for that book, had used the word “psychology” and the phrase “comparative psychology”—46 years prior to Flourens’ *Comparative Psychology—*when he called for the systematic investigation and comparison of instinct and intelligence in all the different mammals (Cuvier, 1808).

Just as Cuvier’s ideas resemble those of twentieth-century ethologists in some ways but not others, the same can be said of his ideas and those of twentieth-century comparative psychologists. Cuvier believed that a science of animal behavior required controlled experiments conducted in lab-like settings, and mid-twentieth-century American comparative psychology certainly fits that description. On the other hand, Cuvier’s aspiration of comparing the mental faculties of all the different mammals was quite the opposite of the American enterprise at mid-century, famously skewered by Frank Beach for its focus on the learning abilities of a single creature, the white rat (Beach, 1950). Since then, and especially since the late twentieth century, comparative psychologists have come to study more species and the techniques they have used have been less invasive, giving the animals in their charge the opportunity to express their species-specific instincts as well as learn from experience (see, for instance, d'Isa et al., 2024).

We suggest that if comparative psychologists wish to identify an early pioneer of their field, Frédéric Cuvier makes a much better choice than Pierre Flourens. Flourens himself would not have pretended otherwise, but the title of his book, *Comparative Psychology*, has led later generations astray.

For the historian of science, the primary challenge is to understand scientific work in its own historical context. Here we have added the perspective of how Cuvier’s work compares to the work of ethologists and comparative ethologists of later periods. We have indicated that his focus on animal behavior, his distinction between instincts and intelligence, and his attention to animal habits, animal training, social behavior and domestication, were the essence of “ethology” as Isidore Geofroy Saint-Hilaire first presented it in the 1850s. On the other hand, modern ethologists would have found his creationism and interest in improving animals for human purposes wholly foreign to them, had they ever encountered his work. As for his qualifications as a comparative psychologist, he explicitly used the phrase “comparative psychology” in connection with his project of comparing instincts and intelligence across the mammalian spectrum, he aimed to reform zoology by making psychology part of it, and he promoted the study of animal behavior as a field deserving of a professorial chair. It would thus seem appropriate for comparative psychologists to think of him as a pioneer of their field, one with an eye to animal psychology’s fit with zoology and with a commitment to using non-invasive methods in his work. As for how Cuvier came to construct the role for himself that he did, this was a function of his particular conditions of existence at an institution where zoologists privileged dead specimens over living ones, but where Cuvier, put in charge of the Museum’s menagerie, sought to create an animal psychology giving zoology a completeness it had previously lacked.

## Data Availability

The original contributions presented in the study are included in the article/supplementary material, further inquiries can be directed to the corresponding author.
